# Rényi and Tsallis Entropies of the Aharonov–Bohm Ring in Uniform Magnetic Fields

**DOI:** 10.3390/e21111060

**Published:** 2019-10-29

**Authors:** Oleg Olendski

**Affiliations:** Department of Applied Physics and Astronomy, University of Sharjah, P.O. Box, Sharjah 27272, UAE; oolendski@sharjah.ac.ae

**Keywords:** quantum ring, Rényi entropy, Tsallis entropy, magnetic field, Aharonov-Bohm effect

## Abstract

One-parameter functionals of the Rényi $R_{\rho ,\gamma }\left(\alpha \right)$ and Tsallis $T_{\rho ,\gamma }\left(\alpha \right)$ types are calculated both in the position (subscript ρ) and momentum (γ) spaces for the azimuthally symmetric 2D nanoring that is placed into the combination of the transverse uniform magnetic field B and the Aharonov–Bohm (AB) flux $\phi_{AB}$ and whose potential profile is modeled by the superposition of the quadratic and inverse quadratic dependencies on the radius *r*. Position (momentum) Rényi entropy depends on the field *B* as a negative (positive) logarithm of $\omega_{eff}\equiv {\left(\omega_{0}^{2}+\omega_{c}^{2}/4\right)}^{1/2}$, where $\omega_{0}$ determines the quadratic steepness of the confining potential and $\omega_{c}$ is a cyclotron frequency. This makes the sum ${R_{\rho }}_{nm}\left(\alpha \right)+{R_{\gamma }}_{nm}\left(\frac{\alpha }{2\alpha -1}\right)$ a field-independent quantity that increases with the principal *n* and azimuthal *m* quantum numbers and satisfies the corresponding uncertainty relation. In the limit α→1, both entropies in either space tend to their Shannon counterparts along, however, different paths. Analytic expression for the lower boundary of the semi-infinite range of the dimensionless coefficient α where the momentum entropies exist reveals that it depends on the ring geometry, AB intensity, and quantum number *m*. It is proved that there is the only orbital for which both Rényi and Tsallis uncertainty relations turn into the identity at α=1/2, which is not necessarily the lowest-energy level. At any coefficient α, the dependence of the position of the Rényi entropy on the AB flux mimics the energy variation with $\phi_{AB}$, which, under appropriate scaling, can be used for the unique determination of the associated persistent current. Similarities and differences between the two entropies and their uncertainty relations are discussed as well.

## 1. Introduction

In an attempt to expand quantum-information theory to the study of the quantum rings (QRs) [1], a recent analysis [2] addressed an influence of the combination of the transverse uniform magnetic field B and the AB flux $\phi_{AB}$ [3] on the position and momentum components of, among others, Shannon entropy [4] of the one-particle orbitals of the flat 2D annulus whose rotationally symmetric potential profile U(r) is modeled in the position of polar coordinates $\left(r,\varphi_{r}\right)$ by the superposition of the quadratic and inverse quadratic dependencies on the radius *r* [5,6,7,8,9,10,11,12,13,14,15]:

(1) $$U\left(r\right)=\frac{1}{2}m^{*}\omega_{0}^{2}r^{2}+\frac{\hbar^{2}}{2m^{*}r^{2}}\;a-\hbar \omega_{0}a^{1/2},$$

where $m^{*}$ is an effective mass of a charge carrier, frequency $\omega_{0}$ defines a steepness of the confining in-plane outer surface of the QR with its characteristic radius $r_{0}={\left[\hbar /\left(2m^{*}\omega_{0}\right)\right]}^{1/2}$, and the positive dimensionless constant *a* describes a strength of the repulsive potential near the origin. General definitions of the Shannon position $S_{\rho }$ and momentum $S_{\gamma }$ quantum-information entropies in the *l*-dimensional spaces read:

(2a) $$\begin{array}{cc} S_{\rho } & =-\int_{R^{l}}\rho \left(r\right)\ln\rho \left(r\right)dr \end{array}$$

(2b) $$\begin{array}{cc} S_{\gamma } & =-\int_{R^{l}}\gamma \left(k\right)\ln\gamma \left(k\right)dk, \end{array}$$

with the integration carried out over the whole available regions where the corresponding waveforms Ψ(r) and Φ(k) that enter into the densities

(3a) $$\begin{array}{cc} \rho (r) & ={\left|\Psi (r)\right|}^{2}, \end{array}$$

(3b) $$\begin{array}{cc} \gamma (k) & ={\left|\Phi (k)\right|}^{2}, \end{array}$$

are defined. Position Ψ(r) and wave vector Φ(k) functions are related to each other through the Fourier transformation, which for the 2D geometry of the QR is expressed as

(4a) $$\begin{array}{c} \Phi_{nm}\left(k,\varphi_{k}\right)=\frac{1}{2\pi }\int_{0}^{2\pi }d\varphi_{r}\int_{0}^{\infty }drr\Psi_{nm}\left(r,\varphi_{r}\right)e^{-ikr\;\cos(\varphi_{r}-\varphi_{k})}, \end{array}$$

(4b) $$\begin{array}{c} \Psi_{nm}\left(r,\varphi_{r}\right)=\frac{1}{2\pi }\int_{0}^{2\pi }d\varphi_{k}\int_{0}^{\infty }dkk\Phi_{nm}\left(k,\varphi_{k}\right)e^{ikr\;\cos(\varphi_{k}-\varphi_{r})}, \end{array}$$

where $\left(k,\varphi_{k}\right)$ are the wave vector polar coordinates and n=0,1,2,…, and m=0,±1,±2,…, are principal and azimuthal indices, respectively. Due to the rotational symmetry of the QR, either dependence is most conveniently represented as a product of the angular and radial parts:

(5a) $$\begin{array}{cc} \Psi_{nm}\;\left(r,\varphi_{r}\right) & =\frac{1}{{\left(2\pi \right)}^{1/2}}e^{im\varphi_{r}}R_{nm}\left(r\right), \end{array}$$

(5b) $$\begin{array}{cc} \Phi_{nm}\;\left(k,\varphi_{k}\right) & =\frac{{\left(-i\right)}^{m}}{{\left(2\pi \right)}^{1/2}}e^{im\varphi_{k}}K_{nm}\left(k\right), \end{array}$$

where the latter ones are

(6a) $$\begin{array}{cc} \; & R_{nm}\left(r\right)=\frac{1}{r_{eff}}\;\;{\left[\frac{n!}{\Gamma (n\;+\;\lambda \;+\;1)}\right]}^{1/2}\;\;\;\;\exp\;\left(\;-\frac{1}{4}\frac{r^{2}}{r_{eff}^{2}}\;\;\right)\;{\left(\;\frac{1}{2}\frac{r^{2}}{r_{eff}^{2}}\;\;\right)}^{\lambda /2}\;\;\;\;L_{n}^{\lambda }\;\left(\;\frac{1}{2}\frac{r^{2}}{r_{eff}^{2}}\;\;\right), \end{array}$$

(6b) $$\begin{array}{cc} \; & K_{nm}\left(k\right)=r_{eff}\;\;{\left[\frac{n!}{\Gamma (n\;+\;\lambda \;+\;1)}\right]}^{1/2}\;\;\;\;\int_{0}^{\infty }\;\;\;e^{-z/2}z^{\lambda /2}L_{n}^{\lambda }\left(z\right)J_{\left|m\right|}\;\left(\;2^{1/2}r_{eff}kz^{1/2}\;\right)dz. \end{array}$$

Here, Γ(x), $L_{n}^{\alpha }\left(x\right)$, and $J_{m}\left(x\right)$ are Γ-function, generalized Laguerre polynomial, and *m*-th order Bessel function of the first kind, respectively [16,17]. In addition,

(7a) $$\begin{array}{cc} r_{eff} & ={\left(\frac{\hbar }{2m^{*}\omega_{eff}}\right)}^{1/2}, \end{array}$$

(7b) $$\begin{array}{cc} \omega_{eff} & ={\left(\omega_{0}^{2}+\frac{1}{4}\omega_{c}^{2}\right)}^{1/2} \end{array}$$

and

(7c) $$\begin{array}{cc} \omega_{c} & =\frac{eB}{m^{*}} \end{array}$$

is the cyclotron frequency,

(7d) $$\begin{array}{cc} \lambda & ={\left(m_{\phi }^{2}+a\right)}^{1/2}, \end{array}$$

(7e) $$\begin{array}{cc} m_{\phi } & =m+\nu , \end{array}$$

with ν being a dimensionless AB flux, i.e., the latter one is expressed in units of the elementary flux quantum $\phi_{0}=h/e$:

(7f) $$\begin{array}{cc} \nu & =\frac{\phi_{AB}}{\phi_{0}}. \end{array}$$

It is easy to check that both function sets are orthonormalized:

(8) $$\begin{array}{cc} \; & \int_{0}^{2\pi }d\varphi_{r}\int_{0}^{\infty }drr\Psi_{n^{\prime }m^{\prime }}^{*}\;\left(r,\varphi_{r}\right)\Psi_{nm}\;\left(r,\varphi_{r}\right)\\ = & \int_{0}^{2\pi }d\varphi_{k}\int_{0}^{\infty }dkk\Phi_{n^{\prime }m^{\prime }}^{*}\;\left(k,\varphi_{k}\right)\Phi_{nm}\;\left(k,\varphi_{k}\right)=\delta_{nn^{\prime }}\delta_{mm^{\prime }}, \end{array}$$

where $\delta_{nn^{\prime }}$ is a Kronecker delta.

It was shown [2] that Shannon position (momentum) quantum information entropy decreases (increases) with the growing field *B* as $\pm 2\ln r_{eff}$, which physically means that in the corresponding space, there is more (less) information about the particle location (intensity of motion). As a result, the sum $S_{\rho }+S_{\gamma }$, describing a total amount of the simultaneous information about the charge carrier, cannot be altered by the uniform magnetic component, and it always satisfies the fundamental restriction [18,19]

(9) $$S_{\rho }+S_{\gamma }\geq l\left(1+\ln\pi \right).$$

In particular, for our geometry, this uncertainty relation becomes tight for the lowest level, n=m=0, of the AB-free $\phi_{AB}=0$, QD, a=0: mathematically, Equations (6) at the zero values of *n*, $\phi_{AB}$, and *a* degenerate to

(10a) $$\begin{array}{cc} {\left.R_{0m}\left(r\right)\right|}_{a=\nu =0} & =\frac{1}{r_{eff}}\frac{1}{{\left(|m|!\right)}^{1/2}}\;{\left(\;\frac{r}{2^{1/2}r_{eff}}\;\;\right)}^{\left|m\right|}\exp\;\left(\;-\frac{1}{4}\frac{r^{2}}{r_{eff}^{2}}\;\;\right), \end{array}$$

(10b) $$\begin{array}{cc} {\left.K_{0m}\left(k\right)\right|}_{a=\nu =0} & =2r_{eff}\frac{1}{{\left(|m|!\right)}^{1/2}}{\left(2^{1/2}r_{eff}k\right)}^{\left|m\right|}\exp\;\left(-r_{eff}^{2}k^{2}\right), \end{array}$$

which means that at a=ν=0, the functions $\Psi_{00}\left(r\right)$ and $\Phi_{00}\left(k\right)$ turn into the 2D Gaussians converting relation (9) into the equality. Next, a dependence of the position entropy $S_{\rho }$ on the normalized AB flux ν strongly resembles that of the energy spectrum:

(11) $$E_{nm}\left(a,\nu ;\omega_{c}\right)=\hbar \omega_{e\;f\;f}\left(2n+\lambda +1\right)+\frac{1}{2}m_{\phi }\hbar \omega_{c}-\hbar \omega_{0}a^{1/2}.$$

Accordingly, the knowledge of $S_{\rho }-\nu$ characteristics permits the calculation of the persistent current [20], which is a negative derivative of the energy with respect to the AB intensity:

(12) $$J_{nm}\equiv -\frac{e}{h}\frac{\partial E}{\partial m}\equiv -\frac{\partial E}{\partial \phi_{AB}}=-\frac{e\omega_{0}}{2\pi }\left[\frac{m_{\phi }}{\lambda }\sqrt{1+\frac{1}{4}{\left(\frac{\omega_{c}}{\omega_{0}}\right)}^{2}}+\frac{1}{2}\frac{\omega_{c}}{\omega_{0}}\right].$$

For many years, physicists and mathematicians have been looking for and discussing generalizations of the Shannon measures from Equations (2). Notably, two of the most famous and frequently used outcomes of these endeavors are one-parameter functionals of the Rényi $R_{\rho ,\gamma }\left(\alpha \right)$ [21,22] (To tell the Rényi entropies from the radial part of the position waveform (Equation (6a)), we will always write the former ones with the subscript ρ or γ denoting a corresponding space.),

(13a) $$\begin{array}{cc} R_{\rho }\left(\alpha \right) & =\frac{1}{1-\alpha }\ln\;\left(\int_{R^{l}}\rho^{\alpha }\left(r\right)dr\right), \end{array}$$

(13b) $$\begin{array}{cc} R_{\gamma }\left(\alpha \right) & =\frac{1}{1-\alpha }\ln\;\left(\;\int_{R^{l}}\gamma^{\alpha }\left(k\right)dk\right), \end{array}$$

and Tsallis $T_{\rho ,\gamma }\left(\alpha \right)$ [23] (or more correctly, Havrda–Charvát–Daróczy–Tsallis [24,25]) types,

(14a) $$\begin{array}{cc} T_{\rho }\left(\alpha \right) & =\frac{1}{\alpha -1}\left(1-\int_{R^{l}}\rho^{\alpha }\left(r\right)dr\right) \end{array}$$

(14b) $$\begin{array}{cc} T_{\gamma }\left(\alpha \right) & =\frac{1}{\alpha -1}\left(1-\int_{R^{l}}\gamma^{\alpha }\left(k\right)dk\right) \end{array}$$

where a non-negative coefficient 0≤α<∞ controls the reaction of the system to its deviation from the equilibrium; namely, the l’Hôpital’s rule deduces that at α→1, both Rényi and Tsallis entropies degenerate to their Shannon counterpart, Equations (2), whereas at vanishingly small magnitudes, this parameter allows equal contributions from the random events of any frequency of actual occurrence, which in the case of the infinite or semi-infinite region of integration in Equations. (13) and (14) leads to the divergence of the corresponding measure (provided it exists) at α→0. On the other hand, extremely strong Rényi or Tsallis parameters pick up in the corresponding probability distributions the global maxima only with a full discard of all other happenings. Using similar arguments, it can be shown that both entropies are decreasing functions of their factor α. Let us mention another particular case of these entropies; namely, Onicescu energies [26], or disequilibria,

(15a) $$\begin{array}{cc} O_{\rho } & =\int_{R^{l}}\rho^{2}\left(r\right)dr, \end{array}$$

(15b) $$\begin{array}{cc} O_{\gamma } & =\int_{R^{l}}\gamma^{2}\left(k\right)dk, \end{array}$$

which describe deviations from the uniform distributions, are expressed with the help of Equations (13) and (14) as

(16) $$O_{\rho ,\gamma }\equiv e^{-R_{\rho ,\gamma }\left(2\right)}\equiv 1-T_{\rho ,\gamma }\left(2\right).$$

For the QR, the position (momentum) Onicescu energy increases (decreases) with the uniform field as $r_{eff}^{-2}$ $\left(r_{eff}^{2}\right)$, which makes the product $O_{\rho }O_{\gamma }$ similar to the sum $S_{\rho }+S_{\gamma }$, a *B*-independent quantity [2].

Sobolev inequality of the conjugated Fourier transforms [27]

(17) $${\left(\frac{\alpha }{\pi }\right)}^{l/(4\alpha )}{\left[\int_{R^{l}}\rho^{\alpha }\left(r\right)dr\right]}^{1/(2\alpha )}\geq {\left(\frac{\beta }{\pi }\right)}^{l/(4\beta )}{\left[\int_{R^{l}}\gamma^{\beta }\left(k\right)dk\right]}^{1/(2\beta )},$$

with the non-negative coefficients α and β obeying the constraint

(18) $$\frac{1}{\alpha }+\frac{1}{\beta }=2,$$

supplemented by the additional requirement

(19) $$\frac{1}{2}\leq \alpha \leq 1,$$

directly establishes the uncertainty relation between the position and momentum Tsallis entropies for each bound quantum orbital [28]:

(20) $${\left(\frac{\alpha }{\pi }\right)}^{l/(4\alpha )}\;\;{\left[1+\left(1-\alpha \right)T_{\rho }\left(\alpha \right)\right]}^{1/(2\alpha )}\geq {\left(\frac{\beta }{\pi }\right)}^{l/(4\beta )}\;\;{\left[1+\left(1-\beta \right)T_{\gamma }\left(\beta \right)\right]}^{1/(2\beta )}.$$

Logarithmization of Equation (17) yields the following inequality for the Rényi components [29,30]:

(21) $$R_{\rho }\left(\alpha \right)+R_{\gamma }\left(\beta \right)\geq -\frac{l}{2}\left(\frac{1}{1-\alpha }\ln\frac{\alpha }{\pi }+\frac{1}{1-\beta }\ln\frac{\beta }{\pi }\right),$$

for which the restriction from Equation (19) is waived. Note that near its unity, the Tsallis parameter turns the corresponding uncertainty, Equation (20), into

(22) $$\frac{1+\left[-2S_{\rho }+l\left(1+\ln\pi \right)\right]\left(\alpha -1\right)/4}{\pi^{l/4}}\geq \frac{1+\left[2S_{\gamma }-l\left(1+\ln\pi \right)\right]\left(\alpha -1\right)/4}{\pi^{l/4}},\;\alpha \to 1,$$

which means that, first, at α=1 it becomes an identity with each of its sides equal to dimensionless $\pi^{-l/4}$ (which follows though directly from Equation (17)), and second, due to the Beckner–Białynicki–Birula–Mycielski inequality [18,19], Equation (9), the relation from Equation (22) turns into the strict inequality at α<1 only, as stated above, Equation (19). At the same time, its Rényi counterpart, Equation (21), with the help of the l’Hôpital’s rule degenerates in the limit α→1 (and, accordingly, β→1) into its Shannon fellow, Equation (9). It was conjectured [31] that the inequalities from Equations (17), (20), and (21) for the lowest-energy level turn into the identities at α=1/2. This issue will be addressed below. An important difference between the entropies is the fact that the Rényi and Shannon functionals are additive (or extensive), whereas the Tsallis dependence is not. More information on each of the entropies can be found in many sources; see, e.g., Refs. [32,33,34,35].

Unique properties of the Rényi and Tsallis entropies explain their wide applications in almost every branch of science and other fields of human acitivity: from seismology [36] and ecology [37,38] with geography [39] through medicine [40,41] and biology [42] to quantum physics [43,44,45,46,47,48,49,50,51,52], free field theories [53,54], and astronomy [55], with many, many others in between and beyond. Partially relevant to our discussion, let us point out that in the latest development, very recent experiments on Bose–Einstein condensate of interacting ${}^{87}$Rb atoms loaded into a 1D [56] or 2D [57] optical lattice and on up to twenty ${}^{40}$Ca${}^{+}$ ions trapped into 1D straight string [58] directly measured the Rényi entanglement entropy with α=2 of these many-body systems. These state-of-the-art achievements open up new perspectives in probing and describing the dynamics of correlated qubits and simultaneously raise new challenges for the correct theoretical description of the Rényi and Tsallis entropies of the miscellaneous quantum structures.

In the present research, a comprehensive description of both measures is provided for the QR with the potential profile described by Equation (1) placed into the superposition of the uniform B and AB $\phi_{AB}$ magnetic fields, with special emphasis being placed on the derivation of the analytic results; for example, even though the expressions for the momentum components of the entropies can, in general, be evaluated numerically only, it is possible to get a simple formula for the lower boundary $\alpha_{TH}$ of the semi-infinite range of the Rényi or Tsallis coefficient at which the integrals in Equations (13b) and (14b) converge. Its inspection reveals that the quantum dot (QD) momentum functionals exist at any non-negative α, whereas for the QR topology, the threshold is determined not only by the potential (or, more precisely, by the antidot strength *a*) but also by the orbital itself. In addition, the AB flux is the only external agent that can control this boundary, since $\alpha_{TH}$ does not depend on *B*. The paths along which both entropies approach their Shannon counterpart at α→1 are shown to be different for the Rényi and Tsallis measures. Limiting cases of the extremely small and infinitely large coefficient α are also addressed. Next, neither the Rényi nor Tsallis uncertainty relation depends on the uniform field B. Since the lowest orbital position $\Psi_{00}\left(r\right)$ and wave vector $\Phi_{00}\left(k\right)$ functions of the AB-free QD (ν=a=0) are described by the 2D Gaussians, the corresponding inequalities, Equations (20) and (21), are saturated for this level at any coefficient α; in particular, for the Tsallis case, a restraint from Equation (19) is waived. The n=m=0 state is a special one also for the QR since it is the only orbital that at α=1/2 turns Equations (20) and (21) into the identities. The dependence of the measures on the AB intensity is also investigated, and it is shown that since the position Rényi entropy at any coefficient α qualitatively repeats the energy dependence on the flux, its knowledge can be useful in predicting the associated persistent currents.

The structure of the research presented below is as follows. Measure properties in the uniform magnetic field are discussed in Section 2, where their position and momentum components are addressed first in Section 2.1 and Section 2.2, respectively, whereas the uncertainty relations are studied in Section 2.3, which is divided into parts devoted to the Tsallis and Rényi functionals. Section 3 contains an analysis of the Rényi entropies’ dependence on the AB flux and its relevance to the prediction of the magnitude of the persistent currents. The discussion is wrapped up in Section 4 with some concluding remarks.

## 2. Entropies in Uniform Magnetic Field B

### 2.1. Position Components

Inserting the forms of the wave functions from Equations (5) and (6) into the general definition of the Rényi, Equations (13), and Tsallis, Equations (14), entropies yields:

(23a) $$\begin{array}{cc} \; & R_{\rho_{nm}}\left(\alpha \right)=2\ln r_{eff}+\ln2\pi ,\\ \; & +\frac{1}{1-\alpha }\ln\left({\left[\frac{n!}{\Gamma (n+\lambda +1)}\right]}^{\alpha }\frac{1}{\alpha^{\alpha \lambda +1}}\int_{0}^{\infty }e^{-z}z^{\alpha \lambda }{\left[L_{n}^{\lambda }{\left(\frac{z}{\alpha }\right)}^{2}\right]}^{\alpha }dz\right), \end{array}$$

(23b) $$\begin{array}{cc} \; & R_{\gamma_{nm}}\left(\alpha \right)=-2\ln r_{eff}+\ln2\pi +\frac{\alpha }{\alpha -1}\ln\frac{n!}{\Gamma (n+\lambda +1)}\\ \; & +\frac{1}{1-\alpha }\ln\int_{0}^{\infty }\;d\xi \xi {\left({\left[\int_{0}^{\infty }\;e^{-z/2}z^{\lambda /2}L_{n}^{\lambda }\left(z\right)J_{\left|m\right|}\;\left(2^{1/2}\xi z^{1/2}\right)\;dz\right]}^{2}\right)}^{\alpha }, \end{array}$$

(24a) $$\begin{array}{cc} \; & T_{\rho_{nm}}\left(\alpha \right)=\frac{1}{\alpha -1}\left(1-\frac{1}{{\left(2\pi r_{eff}^{2}\right)}^{\alpha -1}}\frac{1}{\alpha^{\alpha \lambda +1}}{\left[\frac{n!}{\Gamma (n+\lambda +1)}\right]}^{\alpha }\right.\\ \; & \times \left.\int_{0}^{\infty }e^{-z}z^{\alpha \lambda }{\left[L_{n}^{\lambda }{\left(\frac{z}{\alpha }\right)}^{2}\right]}^{\alpha }dz\right), \end{array}$$

(24b) $$\begin{array}{cc} \; & T_{\gamma_{nm}}\left(\alpha \right)=\frac{1}{\alpha -1}\left[1-{\left(\frac{r_{eff}^{2}}{2\pi }\right)}^{\alpha -1}{\left[\frac{n!}{\Gamma (n+\lambda +1)}\right]}^{\alpha }\right.\\ \; & \times \left.\int_{0}^{\infty }\;d\xi \xi {\left({\left[\int_{0}^{\infty }\;e^{-z/2}z^{\lambda /2}L_{n}^{\lambda }\left(z\right)J_{\left|m\right|}\;\left(2^{1/2}\xi z^{1/2}\right)\;dz\right]}^{2}\right)}^{\alpha }\right]. \end{array}$$

Similar to the Shannon case [2], the whole dependence of the Rényi position and momentum entropies on the uniform magnetic field *B* is embedded in the terms $\pm 2\ln r_{eff}$. Concerning the Tsallis functionals, a dimensional incompatibility of the two items in Equations (24) precludes their direct application for the continuous probability distributions, suggesting instead the forms presented in the corresponding uncertainty relation, Equation (20), but below we will continue to write them, keeping in mind that it is just a formal representation only.

For the ground band, n=0, position components can be evaluated analytically:

(25) $$\begin{array}{cc} R_{\rho_{0m}}\left(\alpha \right) & =2\ln r_{eff}+\ln2\pi +\frac{1}{1-\alpha }\ln\frac{\Gamma (\alpha \lambda +1)}{\alpha^{\alpha \lambda +1}\Gamma^{\alpha }\left(\lambda +1\right)}, \end{array}$$

(26) $$\begin{array}{cc} T_{\rho_{0m}}\left(\alpha \right) & =\frac{1}{\alpha -1}\left[1-\frac{1}{{\left(2\pi r_{eff}^{2}\right)}^{\alpha -1}}\frac{\Gamma (\alpha \lambda +1)}{\alpha^{\alpha \lambda +1}\Gamma^{\alpha }\left(\lambda +1\right)}\right]. \end{array}$$

Three limits of the last two dependencies are

For the Rényi entropy:

(27a) $$\begin{array}{cc} R_{\rho_{0m}}\left(\alpha \right) & =2\ln r_{eff}+\ln2\pi -\ln\alpha ,\\ \; & -\left[\lambda \left(\gamma +\ln\alpha \right)+\ln\left(\alpha \Gamma (\lambda +1)\right)\right]\alpha +\ldots ,\;\alpha \to 0, \end{array}$$

(27b) $$\begin{array}{cc} R_{\rho_{0m}}\left(\alpha \right) & =S_{\rho_{0m}}+\frac{1}{2}\lambda \left[1-\lambda \psi^{\left(1\right)}\left(\lambda \right)\right]\left(\alpha -1\right)+\ldots ,\;\alpha \to 1, \end{array}$$

(27c) $$\begin{array}{cc} R_{\rho_{0m}}\left(\alpha \right) & =2\ln r_{eff}+\ln2\pi +\lambda \left(1-\ln\lambda \right)+\ln\Gamma \left(\lambda +1\right)\\ \; & +\frac{1}{\alpha }\left[\lambda \left(1-\ln\lambda \right)+\ln\Gamma \left(\lambda +1\right)+\frac{1}{2}\ln\frac{\alpha }{2\pi \lambda }\right]+\ldots ,\;\alpha \to \infty , \end{array}$$

and for the Tsallis entropy:

(28a) $$\begin{array}{cc} T_{\rho_{0m}}\left(\alpha \right) & =\frac{2\pi r_{eff}^{2}}{\alpha }-1+\ldots ,\;\alpha \to 0, \end{array}$$

(28b) $$\begin{array}{cc} T_{\rho_{0m}}\left(\alpha \right) & =S_{\rho_{0m}}+c\left(r_{eff},\lambda \right)\left(\alpha -1\right)+\ldots ,\;\alpha \to 1, \end{array}$$

(28c) $$\begin{array}{cc} T_{\rho_{0m}}\left(\alpha \right) & =\frac{1}{\alpha }+\ldots ,\;\alpha \to \infty , \end{array}$$

where the Shannon entropy $S_{\rho_{0m}}$ is [2]

$$S_{\rho_{0m}}=2\ln r_{eff}+\ln2\pi +\ln\Gamma \left(\lambda +1\right)+\lambda \left[1-\psi (\lambda )\right].$$

Here, $\psi \left(x\right)=d\left[\ln\Gamma \left(x\right)\right]/dx=\Gamma^{\prime }\left(x\right)/\Gamma \left(x\right)$ and $\psi^{\left(n\right)}\left(x\right)=d^{n}\psi \left(x\right)/dx^{n}$, n=1,2,… are psi (or digamma) and polygamma functions, respectively [16], and γ is Euler’s constant. In addition, $c(r_{eff},\lambda )$ is a function containing a sum of several terms with miscellaneous products of different powers of $\ln r_{eff}$, λ, Γ(λ), ψ(λ), and $\psi^{\left(1\right)}\left(\lambda \right)$. Due to its unwieldy structure, we do not present its explicit form here. There are a few relevant points worth mentioning during the discussion of these equations. First, at the coefficient α approaching zero, both position entropies diverge (Equations (27a) and (28a)) since, as mentioned in the Introduction, the integration of the constant value over the (semi-)infinite interval essentially yields infinity. Invoking Taylor expansion of Equations (23a) and (24a) with respect to the small parameter α, it is easy to show that the logarithmic- and inverse-like divergences for the Rényi and Tsallis entropies, respectively, are characteristic at α→0 for the arbitrary band with n≥1. Second, a comparison between Equations (27b) and (28b) reconfirms [31] that at the Rényi and Tsallis parameter tending to unity, the corresponding entropies approach their Shannon counterpart along different paths. Next, as it follows, e.g., from Equation (25), at the arbitrary coefficient α and $\phi_{AB}=0$, the position Rényi entropy is an increasing function of the absolute value of the azimuthal index *m*. As our numerical results show, the same statement holds true for the radial quantum number *n* as well. In addition, the leading term of Equation (27c) follows straightforwardly from the expression

(29) $$R_{\rho ,\gamma }\left(\infty \right)=-\ln\;\left(\;\;\;\begin{array}{c} \rho_{max}\\ \gamma_{max} \end{array}\;\;\;\right),$$

with the subscript in the right-hand side denoting a global maximum of the corresponding function. To find its location $r_{max}$ for the position density, one needs to solve a polynomial equation:

(30) $$\left(\lambda -z\right)L_{n}^{\lambda }\left(z\right)-2zL_{n-1}^{\lambda +1}\left(z\right)=0,\;n=0,1,\ldots ,$$

with $z=\frac{1}{2}\frac{r^{2}}{r_{eff}^{2}}$, which for the ground band reproduces the first line of Equation (27c). For an adjacent higher-lying set of levels with n=1, one has $z_{max}=\lambda +\frac{3}{2}-\frac{1}{2}\sqrt{8\lambda +9}$ and

(31) $$R_{\rho_{1m}}\left(\infty \right)=2\ln r_{eff}+\ln2\pi +\ln\Gamma \left(\lambda +2\right)+z_{max}-\lambda \ln z_{max}-2\ln\frac{\sqrt{8\lambda +9}-3}{2}.$$

Finally, as a prerequisite to the analysis of the following subsection, let us underline that position entropies are defined at any positive Rényi or Tsallis parameter.

### 2.2. Momentum Components

For the singly connected geometry of the QD with a=ν=0, the expressions from Equations (25) and (26) simplify to

(32) $$\begin{array}{cc} {\left.R_{\rho_{0m}}\left(\alpha \right)\right|}_{a=\nu =0} & =2\ln r_{eff}+\ln2\pi +\frac{1}{1-\alpha }\ln\frac{\Gamma (|m|\alpha +1)}{{\left(|m|!\right)}^{\alpha }\alpha^{|m|\alpha +1}}, \end{array}$$

(33) $$\begin{array}{cc} {\left.T_{\rho_{0m}}\left(\alpha \right)\right|}_{a=\nu =0} & =\frac{1}{\alpha -1}\left[1-\frac{1}{{\left(2\pi r_{eff}^{2}\right)}^{\alpha -1}}\frac{\Gamma (|m|\alpha +1)}{{\left(|m|!\right)}^{\alpha }\alpha^{|m|\alpha +1}}\right]. \end{array}$$

At the same time, with the help of Equation (10b), the momentum components are expressed analytically as well:

(34) $$\begin{array}{cc} {\left.R_{\gamma_{0m}}\left(\alpha \right)\right|}_{a=\nu =0} & =-2\ln r_{eff}+\ln\frac{\pi }{2}+\frac{1}{1-\alpha }\ln\frac{\Gamma (|m|\alpha +1)}{{\left(|m|!\right)}^{\alpha }\alpha^{|m|\alpha +1}}, \end{array}$$

(35) $$\begin{array}{cc} {\left.T_{\gamma_{0m}}\left(\alpha \right)\right|}_{a=\nu =0} & =\frac{1}{\alpha -1}\left[1-{\left(\frac{2}{\pi }r_{eff}^{2}\right)}^{\alpha -1}\frac{\Gamma (|m|\alpha +1)}{{\left(|m|!\right)}^{\alpha }\alpha^{|m|\alpha +1}}\right]. \end{array}$$

Note that the dependencies of the position and momentum components of, e.g., the Rényi entropy, on the coefficient α are, apart from the constant factor, the same, which can be tracked back to the fact that the corresponding waveforms from Equations (10) present modified Gaussians. This also explains why the sum of the entropies from the corresponding uncertainty relation, Equation (21), takes the same values at the Rényi parameters of one half and infinity (see Section 2.3.2).

Equations (32)–(35) manifest that under these special conditions of the 2D singly connected topology, the momentum entropies exist at any non-negative coefficient α. However, the situation changes drastically at a+|ν|≠0, when the topology turns into the doubly connected one. To derive the lower limit of the semi-infinite range $\left[\alpha_{TH},+\infty \right)$ where the momentum entropies exist, one needs to consider the inner integral in Equations (23b) and (24b), which, as stated before [2], does not have an analytic representation. Nevertheless, for our purpose it suffices to recall that the Laguerre polynomial $L_{n}^{\lambda }\left(z\right)$ of degree n=0,1,2,… is a linear combination of all powers of its argument *z* from zero to *n*. Accordingly, considering the integral

$$\int_{0}^{\infty }\;\;\;e^{-z/2}z^{\lambda /2+n^{\prime }}J_{\left|m\right|}\;\left(\;2^{1/2}\xi z^{1/2}\;\right)dz$$

with $n^{\prime }=0,\ldots n$, one finds [17,59] that it can be represented by the Kummer confluent hypergeometric function ${}_{1}F_{1}\left(a;b;x\right)$ [16,17] as

(36) $$\begin{array}{cc} \; & \int_{0}^{\infty }\;\;\;e^{-z/2}z^{\lambda /2+n^{\prime }}J_{\left|m\right|}\;\left(\;2^{1/2}\xi z^{1/2}\;\right)dz=2^{n^{\prime }+1+\lambda /2}\frac{\Gamma \;\left(n^{\prime }+1+\frac{\lambda +|m|}{2}\right)}{|m|!}\\ \times & \xi^{\left|m\right|}{}_{1}F_{1}\left(n^{\prime }+1+\frac{\lambda +|m|}{2};\left|m\right|+1;-\xi^{2}\right). \end{array}$$

Note that for the AB-free QD, the coefficient λ simplifies to |m|, and then for $n^{\prime }=0$ the Kummer function in Equation (36) degenerates to the fading exponent with $\xi \equiv r_{eff}k$, recovering, in this way, Equation (10b), as expected. In general cases, replacing the Laguerre polynomial in Equations (23b) and (24b) by $z^{n^{\prime }}$, calculating the inner integral with the help of Equation (36), and utilizing asymptotic properties of the confluent hypergeometric function [16], one finds that the outer integrals in the just-mentioned equations will converge [60] at $\alpha >1/(2+\lambda +n^{\prime })$. Consequently, the upper limit of the right-hand side of this inequality, which is achieved at the smallest power of the argument of the Laguerre polynomial, $n^{\prime }=0$, will determine a global range of convergence of the momentum entropies $R_{\gamma }$ and $T_{\gamma }$, and the threshold value is:

(37) $$\alpha_{TH}=\left\{\begin{array}{cc} 0, & a=\nu =0,\\ \frac{1}{2+\lambda }, & a+|\nu |\neq 0. \end{array}\right.$$

Remarkably, this range is not influenced by the uniform field *B* being, on the other hand, a function of the potential profile, as asserted before for the 1D structures [31]. Observe that Equation (37) contains the parameter *a*, defining the inner steepness of U(r) but not the outer confinement that is characterized by $\omega_{0}$. In addition, $\alpha_{TH}$ strongly depends on the orbital itself or, more specifically, on its azimuthal quantum number *m*, which determines the distance from the centre of the ring. In addition, recalling the definition of the parameter λ from Equation (7d), one can use the AB flux as a switch that triggers the existence of the momentum entropies.

Next, using Equation (29) and the fact that for the angle-independent waveforms, m=0, their global maxima are achieved at the zero momentum, k=0, as can be easily shown from Equation (6b), one calculates the corresponding densities as [59]

(38) $$\gamma_{n0}\left(0\right)=r_{eff}^{2}\frac{2^{\lambda +1}}{\pi }\frac{n!}{\Gamma (n\;+\;\lambda \;+\;1)}\frac{\Gamma^{2}\left(\left[\frac{n}{2}\right]+1+\frac{\lambda }{2}\right)}{{\left(\left[\frac{n}{2}\right]!\right)}^{2}},$$

with [x] denoting an integer part of *x*, which leads to the entropies

(39) $$R_{\gamma_{n0}}\left(\infty \right)=-2\ln r_{eff}+\ln\frac{\pi }{2}-\ln\;\left(2^{\lambda }\frac{n!}{\Gamma (n\;+\;\lambda \;+\;1)}\frac{\Gamma^{2}\left(\left[\frac{n}{2}\right]+1+\frac{\lambda }{2}\right)}{{\left(\left[\frac{n}{2}\right]!\right)}^{2}}\right).$$

Note that for the AB-free QD, a=ν=0, when λ in Equation (39) turns to zero, it is consistent at n=0 with the limit α→∞ of Equation (34), as expected.

### 2.3. Uncertainty Relations

Besides playing a fundamental role in the quantum foundations, entropic uncertainty relations find miscellaneous applications in information theory, ranging from entanglement witnessing to wave–particle duality to quantum cryptography, etc. [61,62]. Below, Tsallis and Rényi inequalities are considered separately, but their common features, such as a saturation to identity, are underlined.

#### 2.3.1. Tsallis Entropy

For the ground band, n=0, of the singly connected topology of the QD, a=ν=0, Tsallis inequality from Equation (20), with the help of the dependencies from Equations (33) and (35), is converted to

(40) $${\left(2^{1/2}r_{eff}\right)}^{\frac{1-\alpha }{\alpha }}\frac{\Gamma^{\frac{1}{2\alpha }}\left(|m|\alpha +1\right)}{\alpha^{|m|/2}{\left(\pi |m|!\right)}^{1/2}}\geq {\left(2^{1/2}r_{eff}\right)}^{\frac{\beta -1}{\beta }}\frac{\Gamma^{\frac{1}{2\beta }}\left(|m|\beta +1\right)}{\beta^{|m|/2}{\left(\pi |m|!\right)}^{1/2}},$$

where the coefficients α and β are conjugated by Equation (18). Obviously, due to this, Equation (40) is dimensionally correct, as

(41) $$\frac{1-\alpha }{\alpha }=\frac{\beta -1}{\beta }.$$

Note that for the lowest energy orbital of this configuration, m=0, Equation (40) turns into the identity at any Tsallis parameter α without the restriction from Equation (19), which is explained by the fact that both its position and momentum probability distributions are Gaussian functions, which play a very special role for the entropic inequalities in quantum information [63]. Next, as already mentioned in the Introduction, at any other azimuthal index *m*, the relation from Equation (40) turns into the equality at α=β=1, around which its dimensionless part (without the coefficient $r_{eff}$) becomes

(42) $$\begin{array}{cc} \; & \frac{1+\left(-\ln2-\ln\left(|m|!\right)-\left|m\right|\left[1-\psi (|m|+1)\right]\right)\left(\alpha -1\right)}{\pi^{1/2}}\geq \\ \; & \frac{1+\left(-\ln2+\ln\left(|m|!\right)+\left|m\right|\left[1-\psi (|m|+1)\right]\right)\left(\alpha -1\right)}{\pi^{1/2}},\;\alpha \to 1, \end{array}$$

and since, as it follows from the properties of the psi function [16],

(43) $$\ln\left(|m|!\right)+\left|m\right|\left[1-\psi (|m|+1)\right]>0,\;\left|m\right|\geq 1,$$

the inequality from Equation (42) holds to the left of α=1 only, in accordance with the general Sobolev rule, Equation (19). At the opposite side of this interval, the Tsallis relation simplifies to

(44) $$r_{eff}{\left(\frac{2}{\pi |m|!}\right)}^{1/2}2^{|m|/2}\Gamma \left(\frac{\left|m\right|}{2}+1\right)\geq r_{eff}{\left(\frac{2}{\pi |m|!}\right)}^{1/2}{\left(\frac{\left|m\right|}{e}\right)}^{|m|/2},\;\alpha \to \frac{1}{2},$$

where we have retained the leading terms only in the Taylor expansion of both sides of Equation (40) around α=1/2. The gap between the left and right sides of this inequality widens as the index |m| increases. Moreover, at the extremely large Tsallis parameter, α→∞, the dimensionless parts exchange their places and simultaneously are divided by two, as compared to Equation (44).

Turning to the discussion of the general geometry of the doubly connected topology, a+|ν|>0, let us note first that since here the radius $r_{eff}$ enters either side in the same way as for the QD, Equation (40), the Tsallis inequality at any coefficient α does not depend on the uniform magnetic field B, as was the case for the Shannon regime as well [2]. Next, observe that at α=1/2, the left-hand side of the general Tsallis inequality, Equation (20), becomes

(45) $$\frac{1}{2\pi }\int_{R^{2}}\left|\Psi_{nm}\left(r\right)\right|dr.$$

For the rotationally symmetric orbital (when the function $\Psi_{n0}\left(r\right)$ is real) of the lowest band when the radial component $R_{0m}\left(r\right)$ preserves its sign along the *r* axis), this expression reduces to $\Phi_{00}\left(0\right)$ (see Equation (4a)). On the other hand, in the same limit (i.e., at β=∞), the right-hand sides of Equations (20) and (17) turn to

$$|\Phi_{nm}{\left(k\right)|}_{max}.$$

**Figure 1:**
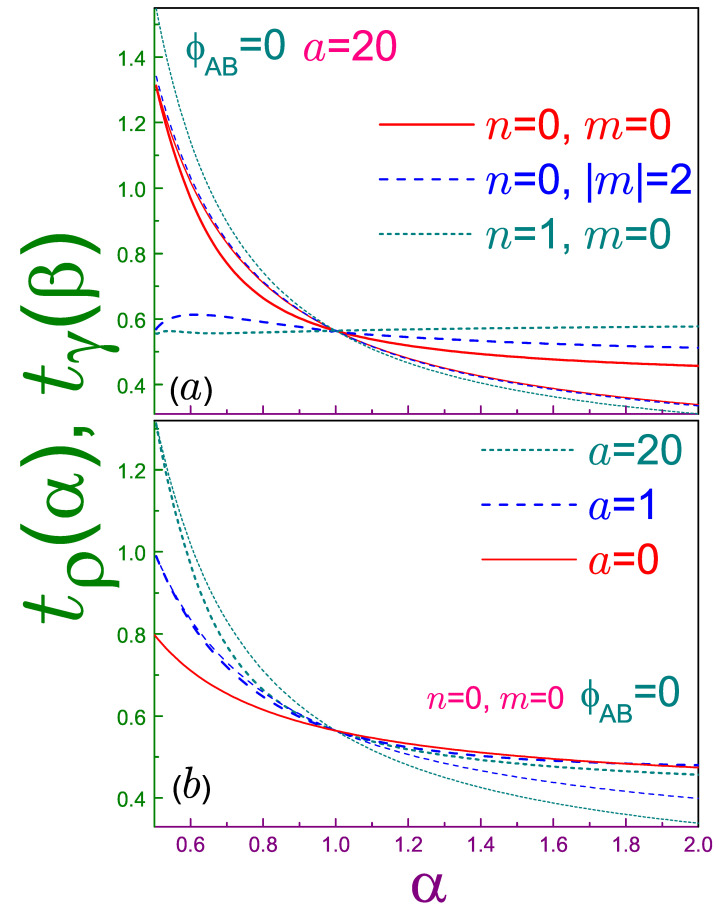
Dimensionless left (thin lines) $t_{\rho_{nm}}\left(\alpha \right)$, Equation (46a), and right (thick curves) $t_{\gamma_{nm}}\left(\beta \right)$, Equation (46b), sides of the Tsallis uncertainty relation, Equation (20), as functions of coefficient α, where in panel (**a**) the parameter *a* is equal to 20, with solid lines showing the n=m=0 state, dashed curves are for the n=0, |m|=2 level, and dotted lines stand for the n=1, m=0 state, whereas window (**b**) depicts the dependencies for several antidot strengths *a* of the n=m=0 orbital: the solid line is for the quantum dot (QD) geometry (a=0), dashed curves are for a=1, and dotted functions are for a=20. Note that vertical ranges in parts (a) and (b) are slightly different. For both panels, the Aharonov–Bohm (AB) flux is zero.

**Figure 2:**
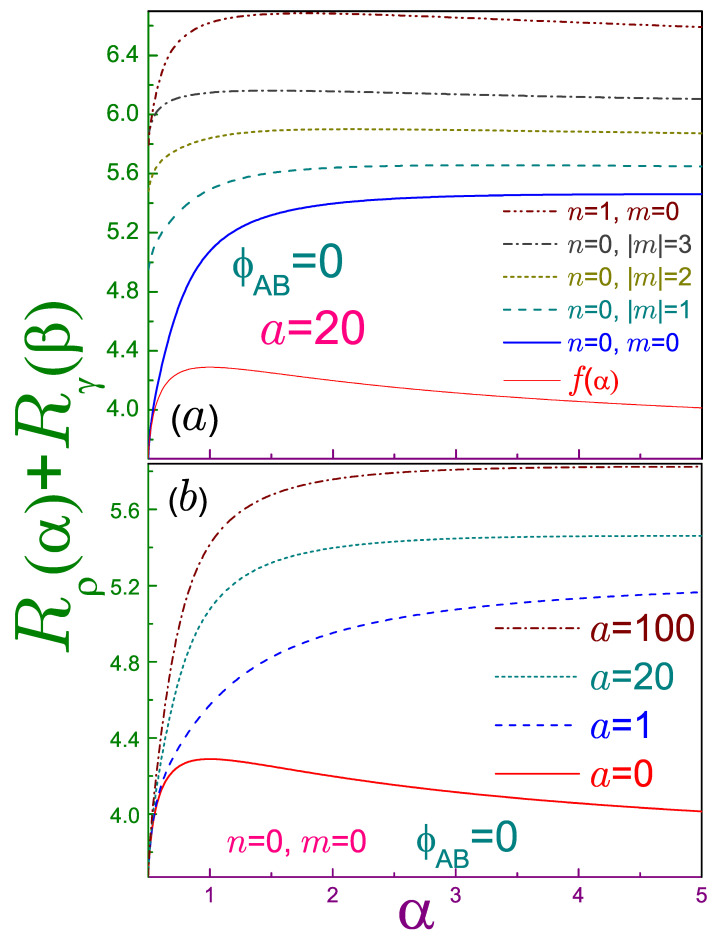
Sum of the position and momentum Rényi entropies $R_{\rho_{nm}}\left(\alpha \right)+R_{\gamma_{nm}}\left(\beta \right)$ as a function of parameter α, where in panel (**a**), the dependencies at the fixed antidot strength a=20 are shown for several indices *n* and *m*, whereas in (**b**), the n=m=0 orbital is depicted at different *a*. In panel (a), the thick solid line is for the n=m=0 level, the dotted curve for the n=0, |m|=1 state, the dashed one stands for the n=0, |m|=2 orbital, the dash-dotted line is the n=0, |m|=3 case, and the dash-dot-dotted dependence describes the n=1, m=0 level, with the thin solid curve representing function f(α) from Equation (48), which is the right-hand side of the Rényi uncertainty relation, Equation (21). The latter dependence is also reproduced by the solid line in panel (b), where the dashed curve is for a=1, the dotted one for a=20 (corresponding to the thick solid line in panel (a), and the dash-dotted curve is for a=100. For both windows, the AB intensity is zero, $\phi_{AB}=0$, and their upper vertical limits differ from each other.

As already mentioned in Section 2.2, for the angle-independent, m=0, momentum functions, their global maximum is located at the zero wave vector. Hence, we have shown that the n=m=0 orbital at α=1/2 transforms the Tsallis inequality into the identity. The existence of such a level was conjectured before [31], when it was stated, however, that it has to be the lowest energy state. But the well-known property of the QR is the fact that the increasing magnetic field B causes consecutive crossings of the energies of the same band orbitals with adjacent non-positive azimuthal indices [6,7,11,13]; for example, the n=m=0 level exhibits the lowest energy only in the range of the cyclotron frequencies from zero to [13]

$$2^{1/2}\frac{{\left(a+1\right)}^{1/2}-a^{1/2}}{{\left({\left[a\left(a+1\right)\right]}^{1/2}-a\right)}^{1/2}}\;\omega_{0},$$

after which it lies above the n=0, m=−1 state. Accordingly, the previous conjecture [31] stays correct in the sense that there is the only orbital that at α=1/2 saturates the Tsallis uncertainty relation; however, it is not necessarily the lowest energy level (at least, for the 2D structures in the magnetic field). Solid lines in panel (a) of Figure 1, which depicts the quantities

(46a) $$\begin{array}{cc} t_{\rho }\left(\alpha \right) & =r_{eff}^{\frac{\alpha -1}{\alpha }}{\left(\frac{\alpha }{\pi }\left[1+\left(1-\alpha \right)T_{\rho }\left(\alpha \right)\right]\right)}^{1/(2\alpha )}, \end{array}$$

(46b) $$\begin{array}{cc} t_{\gamma }\left(\beta \right) & =r_{eff}^{\frac{1-\beta }{\beta }}{\left(\frac{\beta }{\pi }\left[1+\left(1-\beta \right)T_{\gamma }\left(\beta \right)\right]\right)}^{1/(2\beta )}, \end{array}$$

which are dimensionless left and right parts, respectively, of Equation (20), emphasize the saturation by the n=m=0 quantum state of the corresponding uncertainty not only at α=1, as all other orbitals do (see dashed and dotted curves), but at the Tsallis coefficient being equal to one half as well. Window (b) compares the influence of the width of the ring on the interrelation between position and momentum Tsallis parts of this orbital: It can be seen that for the thinner ring (greater *a* [2,13]), the difference between them increases in the interval from Equation (19). The dependencies shown in this figure as well as in Figure 2 are universal in the sense that they do not depend on the uniform magnetic field. For completeness, we also provide the analytic expression of the left-hand side of the Tsallis inequality for the ground band, n=0:

(47) $${\left(2^{1/2}r_{eff}\right)}^{\frac{1-\alpha }{\alpha }}\frac{\Gamma^{\frac{1}{2\alpha }}\left(\lambda \alpha +1\right)}{\pi^{1/2}\alpha^{\lambda /2}\Gamma^{\frac{1}{2}}\left(\lambda +1\right)},$$

which generalizes its QD counterpart from Equation (40).

#### 2.3.2. Rényi Entropy

As follows from Equations (23), the uncertainty relation, Equation (21), is not affected by the uniform magnetic field. This statement, similar to its Tsallis counterpart from the previous subsection, expands to any Rényi parameter a previous conclusion for the Shannon entropies [2].

Equations (32) and (34) with m=0 directly show that the AB-free QD lowest energy orbital saturates the entropic inequality at the arbitrary coefficient α. The explanation for this is the same as for the Tsallis entropy (see Section 2.3.1).

For l=2, the right-hand side of inequality (21), which we will denote as

(48) $$f\left(\alpha \right)=2\left[\ln\pi -\ln\alpha +\frac{\alpha -1/2}{\alpha -1}\ln\left(2\alpha -1\right)\right],$$

reaches its only maximum of 2(1+lnπ)=4.2894… at the Shannon regime, α=1, and approaches 2ln2π=3.6757… at α→1/2 and α→∞ [31]. For an arbitrary *m*, the same limits of the sum $R_{\rho_{0m}}\left(\alpha \right)+R_{\gamma_{0m}}\left(\beta \right)$ at a=ν=0 are

(49a) $$\begin{array}{cc} \; & 2\ln2\pi +|m|\left(1+\ln2\right)+\ln\frac{\Gamma^{2}\;\left(\frac{\left|m\right|}{2}+1\right)}{{\left|m\right|}^{\left|m\right|}}-2\left(\alpha -\frac{1}{2}\right)\ln\left(\alpha -\frac{1}{2}\right)+\ldots ,\;\alpha \to \frac{1}{2}, \end{array}$$

(49b) $$\begin{array}{cc} \; & 2(1+\ln\pi +\ln(|m|!)+|m|\left[1-\psi (|m|+1)\right]),\\ \; & -\left[\frac{1}{3}+\frac{1}{3}{\left|m\right|}^{3}{\psi (2,|m|+1)+|m|}^{2}\psi \left(1,|m|+1\right)-\frac{2}{3}\left|m\right|\right]{\left(\alpha -1\right)}^{2}+\ldots ,\;\alpha \to 1, \end{array}$$

(49c) $$\begin{array}{cc} \; & 2\ln2\pi +|m|\left(1+\ln2\right)+\ln\frac{\Gamma^{2}\;\left(\frac{\left|m\right|}{2}+1\right)}{{\left|m\right|}^{\left|m\right|}}+\frac{1}{2}\frac{\ln\alpha }{\alpha }+\ldots ,\;\alpha \to \infty . \end{array}$$

Note that, as follows from Equations (49a) and (49c), the sum of the entropies of the generalized Gaussian approaches its edge values (which are equal to each other due to the fact that each item in it has the same dependence on the Rényi parameter and, as a result and due to the condition from Equation (18), at the rims α and β simply interchange their places) from above, and since the expression in the square brackets in Equation (49b) is always positive, the left-hand side of Equation (21) reaches its maximum at the Shannon entropy. In addition, the leading terms in all three cases are increasing functions of the magnetic index, which means that at a greater |m|, the corresponding curve lies higher, satisfying, of course, the uncertainty relation. As our numerical results show, the same statement holds true at a fixed quantum number *m* and an increasing principal index *n*.

For the QR, a>0, a comparison of Equations (25), (39), and (48) proves that the n=m=0 orbital converts the Rényi uncertainty into the identity at α=1/2, as it did for the Tsallis inequality as well. This is also exemplified in Figure 2a, which shows that its sum $R_{\rho }\left(\alpha \right)+R_{\gamma }\left(\beta \right)$ at any parameter α is the smallest one, as compared to other levels. Dependence of the left-hand side of Equation (21) on *n* and |m| is the same as for the QD described in the previous paragraph. Contrary to Equations (49a) and (49c), for the doubly connected topology the sum approaches different limits at the Rényi parameters one half and infinity. The location of the only (relatively broad, as compared to the QD) maximum of $R_{\rho_{nm}}\left(\alpha \right)+R_{\gamma_{nm}}\left(\beta \right)$ is now *n*- and |m|-dependent: as panel (a) demonstrates, it is shifted to smaller α at greater |m| and *n*. The same effect is achieved by thinning the ring, as depicted in window (b) of Figure 2, where it is also shown that the sum gets larger for an increasing antidot strength. In addition, it is seen that the transformation of the uncertainty relation for the n=m=0 state into the identity at α=1/2 is independent from the nonzero *a*, as follows from Equations (25) and (39). Finally, the remark about the conjecture [31] discussed for the Tsallis entropies in Section 2.3.1 directly applies to their Rényi counterparts too.

## 3. AB Rényi Entropy

Due to the dimensional incompatibility of the continuous distributions of the two items in the right-hand sides of Equations (14), we do not discuss dependencies of the Tsallis measures on $\phi_{AB}$. To describe a variation of the position Rényi entropy $R_{\rho_{00}}\left(\alpha \right)$ with the AB flux, one has to calculate Taylor expansion of Equation (25) with respect to the parameter ν and truncate the series at the first nonvanishing power of the AB intensity:

(50) $$\begin{array}{cc} R_{\rho_{00}}\left(\nu ;\alpha \right)= & 2\ln r_{eff}+\ln2\pi +\frac{1}{\alpha -1}\left[\left(a^{1/2}\alpha +1\right)\ln\alpha +\ln\frac{\Gamma^{\alpha }\;\left(a^{1/2}+1\right)}{\Gamma \;\left(a^{1/2}\alpha +1\right)}\right]\\ + & \frac{\alpha }{2a^{1/2}\left(\alpha -1\right)}\left[\psi \left(a^{1/2}+1\right)-\psi \left(a^{1/2}\alpha +1\right)+\ln\alpha \right]\nu^{2}. \end{array}$$

Properties of the digamma function [16] applied to the analysis of the term at $\nu^{2}$ reveal that the entropy $R_{\rho_{00}}$ at an arbitrary Rényi parameter and width of the ring is similar to the zero-uniform-field energy

(51) $$E_{n0}\left(a,\nu ;0\right)=\hbar \omega_{0}\;\left(\;2n+1+\frac{1}{2a^{1/2}}\nu^{2}\right),$$

a convex function of the flux, and since the persistent current is expressed with the help of the derivative of the energy with respect to ν, Equation (12), the position entropy can be used for evaluating $J_{nm}$ too. A steepness $\partial R_{\rho_{nm}}\left(\nu ;\alpha \right)/\partial \nu$ of the $R_{\rho }-\nu$ characteristics is strongly α− and a−dependent as, for example, three important limits show:

(52a) $$\begin{array}{cc} \; & R_{\rho_{00}}\left(\nu ;\alpha \right)=2\ln r_{eff}+\ln2\pi -\ln\alpha -\left[a^{1/2}\left(\gamma +\ln\alpha \right)+\ln\left(\alpha \Gamma \left(a^{1/2}+1\right)\right)\right]\alpha \\ \; & -\frac{1}{2a^{1/2}}\left[\gamma +\psi \left(a^{1/2}+1\right)+\ln\alpha \right]\alpha \nu^{2},\;\alpha \to 0, \end{array}$$

(52b) $$\begin{array}{cc} \; & R_{\rho_{00}}\left(\nu ;\alpha \right)=2\ln r_{eff}+\ln2\pi +\ln\;\Gamma \;\left(a^{1/2}+1\right)+a^{1/2}\left[1-\psi \;\left(a^{1/2}\right)\right]\\ \; & +\frac{1}{2}a^{1/2}\left[1-a^{1/2}\psi^{\left(1\right)}\left(a^{1/2}\right)\right]\left(\alpha -1\right)+\frac{1}{2}\left[\frac{1}{a^{1/2}}-\psi^{\left(1\right)}\left(a^{1/2}+1\right)\right]\nu^{2}\\ \; & +\left[\frac{1}{2}+\frac{3}{a^{1/2}}+a\psi^{\left(2\right)}\left(a^{1/2}\right)-a^{1/2}\psi^{\left(1\right)}\left(a^{1/2}\right)\right]\frac{\alpha -1}{a^{1/2}}\nu^{2},\;\alpha \to 1, \end{array}$$

(52c) $$\begin{array}{cc} \; & R_{\rho_{00}}\left(\nu ;\alpha \right)=2\ln r_{eff}+\ln2\pi +a^{1/2}\left(1-\ln a^{1/2}\right)+\ln\Gamma \left(a^{1/2}+1\right)\\ \; & +\frac{1}{\alpha }\left[a^{1/2}\left(1-\ln a^{1/2}\right)+\ln\Gamma \left(a^{1/2}+1\right)+\frac{1}{2}\ln\frac{\alpha }{2\pi a^{1/2}}\right]\\ \; & +\frac{\psi \left(a^{1/2}\;+\;1\right)\;-\;\ln a^{1/2}}{2a^{1/2}}\nu^{2}+\left[\frac{1}{2a^{1/2}}\;+\;\psi \;\left(a^{1/2}\;\right)-\ln a^{1/2}\right]\frac{\nu^{2}}{\alpha },\;\alpha \to \infty . \end{array}$$

**Figure 3:**
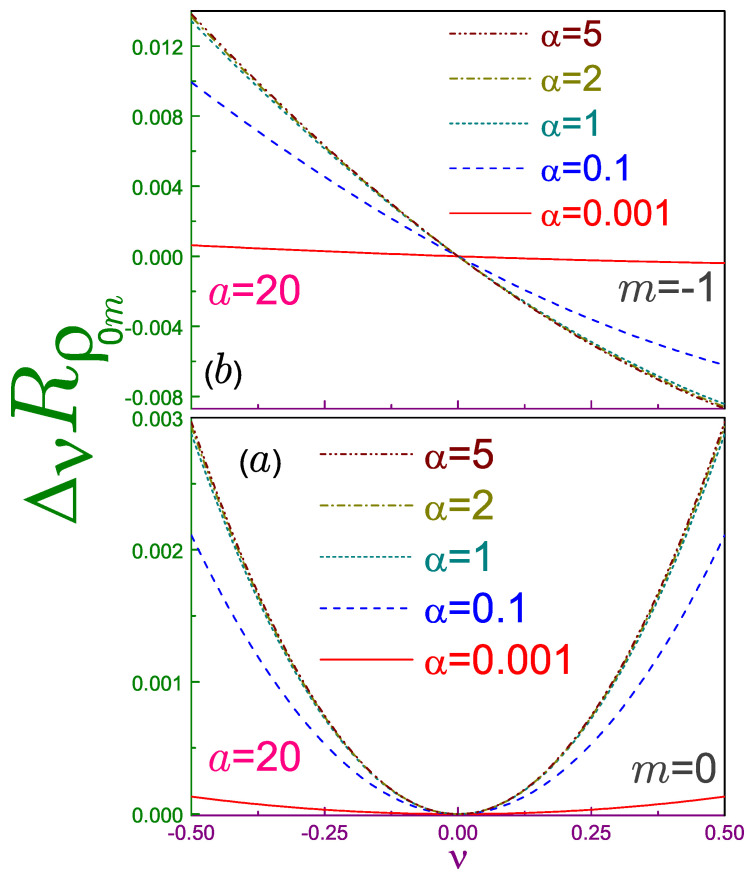
Difference $\Delta_{\nu }R_{\rho_{0m}}$, Equation (53), at a=20, B=0, $r_{0}=1$ and (**a**) m=0, and (**b**) m=−1, where solid lines are for the Rényi parameter being equal to 0.001, dashed curves are for α=0.1, dotted ones are for α=1, dash-dotted lines are for α=2, and dash-dot-dotted curves depict the dependence at α=5.

First, let us point out that at α=1, the Rényi entropy, Equation (52b), turns into its Shannon counterpart [2] (Equation (38) in [2] contains two typos: first, the free item “+1” on the upper line of its right-hand side should be dropped, and second, the argument of the function $\psi^{\left(1\right)}$ on the third line should be $a^{1/2}$ instead of *a*. In addition, the item $\frac{1}{2}$ on the first line of Equation (40) there should enter with the negative sign. These typos do not affect any other results presented in that paper.), as expected. Second, a dying coefficient α leads not only to the logarithmic divergence of the entropy but simultaneously suppresses its dependence on the AB field, as Equation (52a) demonstrates. To exemplify a variation in the speed of change of the entropy with flux $\partial R_{\rho_{00}}/\partial \nu$ at different Rényi coefficients, Figure 3a depicts the quantity

(53) $$\Delta_{\nu }R_{\rho_{nm}}=R_{\rho_{nm}}\left(\nu ;\alpha \right)-R_{\rho_{nm}}\left(0;\alpha \right)$$

at a=20 for n=m=0. It is seen that as the parameter α decreases to extremely small values (eventually reaching zero), the entropy loses its dependence on the flux (eventually becoming completely flat). This has a clear physical explanation; namely, at a vanishing α, the integrand in Equation (13a) degenerates to unity, which is not affected by the variation of the AB field. Increasing the Rényi coefficient makes the slope steeper, and at α≳1, the $R_{\rho }-\nu$ curve practically does not depend on α, as a comparison of the dotted, dash-dotted, and dash-dot-dotted lines in the figure reveals. This slope saturation can also be deduced from the analysis of the corresponding terms

$$\frac{1}{2}\left[\frac{1}{a^{1/2}}-\psi^{\left(1\right)}\left(a^{1/2}+1\right)\right]$$

and

$$\frac{\psi \left(a^{1/2}\;+\;1\right)\;-\;\ln a^{1/2}}{2a^{1/2}}$$

at $\nu^{2}$ in Equations (52b) and (52c), respectively: they almost do not differ from each other, especially at moderate and large *a*s. Let us also point out that the convexity of the Rényi entropy and the relation between $R_{\rho ,\gamma }$ and Onicescu energy, Equation (16), explains the concavity of the position component of the latter [2].

**Figure 4:**
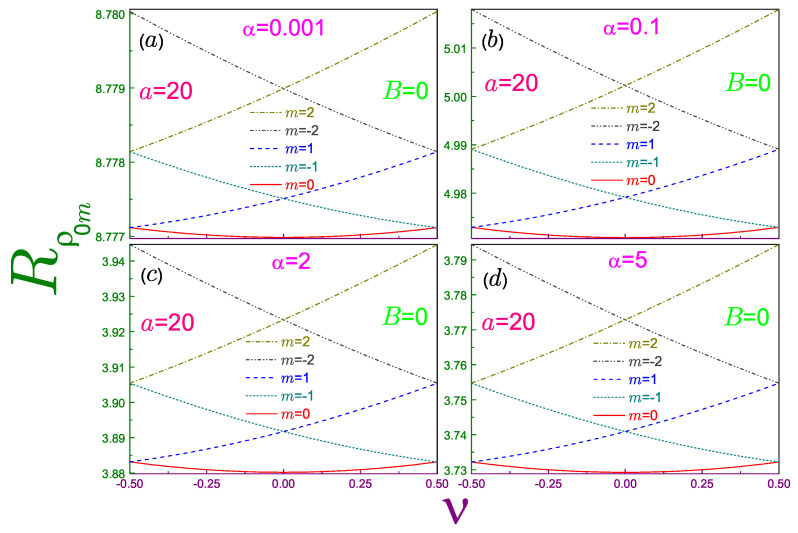
Position Rényi entropies $R_{\rho_{0m}}$ as functions of the normalized AB flux ν at a=20, zero magnetic field, and several parameters α, where panel (**a**) is for α=0.001, window (**b**) is for α=0.1, subplot (**c**) is for α=2, and panel (**d**) shows the entropies at α=5. In each of the windows, a solid curve denotes the orbital with m=0, the dotted line is for the level with m=−1, the dashed line is for m=1, the dash-dotted line describes the entropy of the state with m=−2, and the dash-dot-dotted curve with m=2. Radius $r_{0}$ is assumed to be equal to unity. Note different scales and ranges of the vertical axis in each of the panels.

Figure 4 shows $R_{\rho_{0m}}-\nu$ characteristics for the three smallest |m| and several Rényi parameters. A corresponding analysis of the Shannon dependencies revealed a strong similarity between $R_{\rho }\left(\nu ;1\right)$ and the energy spectrum [2]. This resemblance survives qualitatively at the arbitrary coefficient α≠1; in particular, relations

(54a) $$\begin{array}{cc} R_{\rho_{nm}}\;\left(-\frac{1}{2};\alpha \right) & =R_{\rho_{n,-m+1}}\;\left(-\frac{1}{2};\alpha \right), \end{array}$$

(54b) $$\begin{array}{cc} R_{\rho_{nm}}\;\left(\frac{1}{2};\alpha \right) & =R_{\rho_{n,-m-1}}\;\left(\frac{1}{2};\alpha \right), \end{array}$$

which are elementary derived from Equation (23a), are an exact replica of the corresponding degeneracy of the energy spectrum in the zero uniform magnetic field [2]. This is a consequence of the invariance of the radial part of the position waveform (Equation (6a)), energy (Equation (11)), and persistent current (Equation (12)), under the transformation

(55) m→m−1,ν→ν+1.

In addition, at any α the slope retains the same sign as the azimuthal index *m*. Quantitatively, the magnitude of the steepness $|\partial R_{\rho_{nm}}/\partial \nu |$ for any orbital, similar to the n=m=0 state, decreases as the Rényi coefficient tends to progressively smaller values, eventually becoming perfectly flat at α=0, whereas at α≳1, it is almost not affected by the variation of this parameter. Figure 3b shows both of these features for the n=0, m=−1 level. One can say that a decreasing Rényi factor increases the density of the position components, and with its lowest threshold moving higher and in the opposite regime of the huge α, the number of the position Rényi entropies per unit interval saturates, its bottom being determined by the antidot strength *a*.

Discussing momentum entropy dependence on the AB field, one has to recall that there is a lower nonzero threshold at which $R_{\gamma_{nm}}$ can be calculated. Equation (37) reveals that if the momentum component, e.g., for the rotationally symmetric orbitals, m=0, takes a finite value at the zero flux, it will stay bounded at any arbitrary $\phi_{AB}$. However, the opposite is not always true: a decreasing AB intensity increases for these levels $\alpha_{TH}$, which can lead to the divergence of the corresponding entropy at a fixed Rényi coefficient. For m≠0 states, the symmetry with respect to the sign of the flux is lost; accordingly, the entropy that was finite at some particular α and zero AB field can become infinite with the variation of the flux. Thus, as mentioned in Section 2.2, the AB intensity can switch the existence of the momentum functionals.

**Figure 5:**
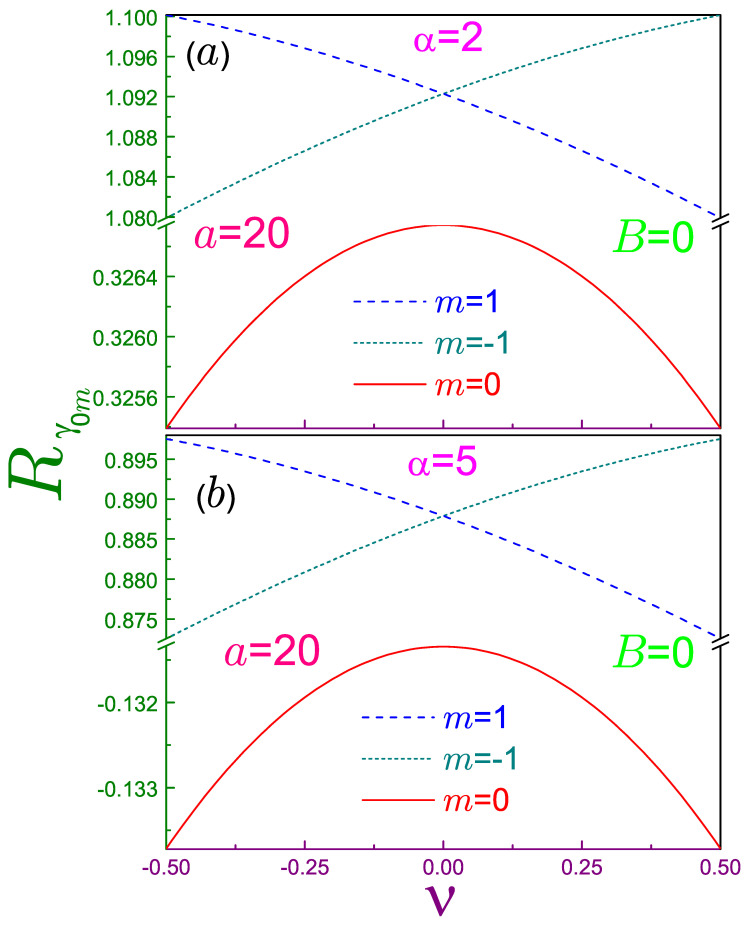
Momentum Rényi entropies $R_{\gamma_{0m}}$ for m=0 and ±1 as functions of the normalized AB flux ν for (**a**) α=2 and (**b**) α=5. All other parameters and conventions are the same as in Figure 4. Due to the relatively small change in the entropies as compared to the distance between $R_{\gamma_{00}}$ and $R_{\gamma_{0,\pm 1}}$, vertical axes breaks have been inserted in panel (a) from 0.3267 to 1.0799 and in window (b) from −0.13133 to 0.8726. Also note the different scales above and below the break in subplot (b).

Numerical analysis shows that momentum components $R_{\gamma_{n0}}$, contrary to their position counterparts, are concave functions of the flux. A particular case of this statement for the Shannon entropy, α=1, was established before [2] and is generalized here to all other values of the Rényi coefficient. Figure 5 exemplifies the entropy behavior at the two parameters α. Steepness $|\partial R_{\gamma_{nm}}/\partial \nu |$ becomes more precipitous for larger α, as was the case for $R_{\rho_{nm}}$ as well. It is observed that for the same orbital, the sign of the slope of the momentum Rényi functional is just the opposite to its position fellow. The relations similar to Equations (54) do not exist for $R_{\gamma_{nm}}$, which is a direct consequence of the expression for the corresponding radial waveform, Equation (6b). The gap between the entropies with different |m| gets wider as the Rényi factor grows, whereas the range of change of each $R_{\gamma_{nm}}$ at α≳1 stays almost unchanged. This is the reason the vertical breaks have been introduced in Figure 5.

As a last note of this discussion, let us mention that, similar to the Shannon case [2], the background uniform magnetic field, B≠0, does not change the shape of the $R_{\rho ,\gamma }-\phi_{AB}$ characteristics but simply shifts them in the vertical direction, as follows, for instance, from Equation (50). Accordingly, Equations (54) representing the invariance under the transformation from Equation (55) stay intact too. A structure of the energy spectrum in this case is analyzed in [2].

## 4. Conclusions

Knowledge of the Rényi and Tsallis entropies is important in studying various phenomena in many branches of science. This general fact was confirmed above by showing that, for example, the Rényi position components of the QR at any coefficient α qualitatively repeat the behavior of the AB energy spectrum in zero uniform magnetic fields, which can be used for predicting the magnitude of the associated persistent currents. Among other findings, let us mention the equation for the lowest boundary of the dimensionless Rényi/Tsallis coefficient at which the corresponding momentum components exist, Equation (37), which shows that there is an abrupt jump when the topology of the structure changes from the singly to the doubly connected one. Note that for the orbitals with position densities concentrated far away from the origin (which mathematically means that a≫1 and/or |m|≫1), the threshold from Equation (37) asymptotically approaches that of the QD, which is physically explained by the negligible influence of the inner confining potential on their properties. Uncertainty relations for both entropies are independent of the uniform field *B* and become tight not only for the 2D Gaussians of the lowest QD orbital, (Equations (10) with m=0, but also turn into the identity at α=1/2 for the QR n=m=0 level, which is the only state that reaches this saturation. In this way, earlier conjecture [31] about the uniqueness of this orbital that should have the lowest energy is amended since the well-known property of the QR energy spectrum is crossings of the levels as the field *B* increases.

Flexibility of the model described by the potential from Equation (1) leads to miscellaneous limiting geometries [2,6,7]; in particular, keeping constant the radius $r_{min}=2^{1/2}a^{1/4}r_{0}$ at which the sole zero minimum of U(r) is achieved and simultaneously unrestrictedly enlarging $\omega_{0}$, one arrives at the 1D ring of the same radius $r_{min}$ pierced by the total magnetic flux $\phi_{tot}=\pi r_{min}^{2}B+\phi_{AB}$ [64,65,66,67] when the position waveform, Equation (5a), energy spectrum, Equation (11), and persistent current, Equation (12), degenerate, respectively, to

(56a) $$\begin{array}{cc} \Psi_{m}\left(\varphi_{r}\right) & =\frac{1}{{\left(2\pi \right)}^{1/2}}e^{im\varphi_{r}}, \end{array}$$

(56b) $$\begin{array}{cc} E_{m}\left(\theta \right) & =\frac{\hbar^{2}}{2m^{*}r_{min}^{2}}{\left(m+\theta \right)}^{2}, \end{array}$$

(56c) $$\begin{array}{cc} J_{m}\left(\theta \right) & =-\frac{e\hbar }{m^{*}r_{min}^{2}}\left(m+\theta \right), \end{array}$$

with $\theta =\phi_{tot}/\phi_{0}$. Observe that due to the frozen radial motion, the principal quantum index *n* has been dropped from Equations (56). Since $\Psi_{m}\left(\varphi_{r}\right)$ and ${\left[2m^{*}E_{m}\left(\theta \right)\right]}^{1/2}$ describe the eigenstates of the angular momentum of this AB rotator, the corresponding Rényi uncertainty relation is saturated by them and does not depend on α and β [29]. Let us also note that this model can apparently be used as a foundation of the quantum-informational analysis of the relevant more complicated structures, such as, for example, nanohelices [68,69,70,71].

Armed with the expression for the Rényi entropies, one can build up shape Rényi complexities [72]:

(57) $$C_{\rho ,\gamma }\left(\alpha \right)=e^{R_{\rho ,\gamma }\left(\alpha \right)}O_{\rho ,\gamma },$$

where the formulas for the disequilibria $O_{\rho ,\gamma }$ are given in Equations (15). For example, this was very recently done for a noncommutative anisotropic oscillator in a homogeneous magnetic field [73]. Regarding this dimensionless quantity, let us just point out that for our geometry, neither its position $C_{\rho }$ or its wave vector $C_{\gamma }$ component depends on the uniform intensity *B*.

Finally, let us remark that above, the Rényi and Tsallis functionals were considered in the position and momentum spaces, which are two non-commuting observables. In the last year or so, Rényi [74,75] and Tsallis [75] entropies were proposed in energy and time domains; in particular, corresponding uncertainty relations were derived [74,75]. Application of these measures and associated inequalities to the analysis of the QDs and QRs may present an interesting development of quantum information and quantum cryptography protocols.

## Abbreviations

The following abbreviations are used in this manuscript:

AB	Aharonov-Bohm
*n*D	*n*-dimensional
QD	Quantum dot
QR	Quantum Rring

## References

[1] Fomin V.M. (2014). Physics of Quantum Rings.

[2] Olendski O. (2019). Quantum information measures of the Aharonov–Bohm ring in uniform magnetic fields. Phys. Lett. A.

[3] Aharonov Y., Bohm D. (1959). Significance of electromagnetic potentials in the quantum theory. Phys. Rev..

[4] Shannon C.E. (1948). A mathematical theory of communication. Bell Syst. Tech. J..

[5] Bogachek E.N., Landman U. (1995). Edge states, Aharonov-Bohm oscillations, and thermodynamic and spectral properties in a two-dimensional electron gas with an antidot. Phys. Rev. B.

[6] Tan W.-C., Inkson J.C. (1996). Landau quantization and the Aharonov-Bohm effect in a two-dimensional ring. Phys. Rev. B.

[7] Tan W.-C., Inkson J.C. (1996). Electron states in a two-dimensional ring-an exactly soluble model. Semicond. Sci. Technol..

[8] Tan W.-C., Inkson J.C. (1999). Magnetization, persistent currents, and their relation in quantum rings and dots. Phys. Rev. B.

[9] Fukuyama H., Sasaki T., Yokoyama K., Ishikawa Y. (2002). Orbital magnetism in two-dimensional systems. J. Low Temp. Phys..

[10] Bulaev D.V., Geyler V.A., Margulis V.A. (2004). Effect of surface curvature on magnetic moment and persistent currents in two-dimensional quantum rings and dots. Phys. Rev. B.

[11] Simonin J., Proetto C.R., Barticevic Z., Fuster G. (2004). Single-particle electronic spectra of quantum rings: A comparative study. Phys. Rev. B.

[12] Margulis V.A., Mironov V.A. (2008). Magnitnyi moment kol’ca Volkano. Fiz. Tverd. Tela (S.-Peterburg).

[13] Olendski O., Barakat T. (2014). Magnetic field control of the intraband optical absorption in two-dimensional quantum rings. J. Appl. Phys..

[14] Xiao M., Reyes-Serrato A. (2016). Analytic Aharonov-Bohm rings: currents readout from Zeeman spectrum. Int. J. Mod. Phys. B.

[15] Negrete O.A., Peña F.J., Vargas P. (2018). Magnetocaloric effect in an antidot: the effect of the Aharonov-Bohm flux and antidot radius. Entropy.

[16] Abramowitz M., Stegun I.A. (1964). Handbook of Mathematical Functions.

[17] Gradshteyn I.S., Ryzhik I.M. (2014). Table of Integrals, Series, and Products.

[18] Białynicki-Birula I., Mycielski J. (1975). Uncertainty relations for information entropy in wave mechanics. Commun. Math. Phys..

[19] Beckner W. (1975). Inequalities in Fourier analysis. Ann. Math..

[20] Büttiker M., Imry Y., Landauer R. (1983). Josephson behavior in small normal one-dimensional rings. Phys. Lett. A.

[21] Rényi A., Neyman J. (1961). On Measures of Entropy and Information. Contributions to the Theory of Statistics, Proceedings of the Fourth Berkeley Symposium on Mathematical Statistics and Probability, University of California: Davis, CA, USA, 20 June–30 July 1960.

[22] Rényi A. (1970). Probability Theory.

[23] Tsallis C. (1988). Possible generalization of Boltzmann-Gibbs statistics. J. Stat. Phys..

[24] Havrda J., Charvát F. (1967). Quantification method of classification processes. Concept of structural *a*-entropy. Kybernetika.

[25] Daróczy Z. (1970). Generalized information functions. Inform. Control.

[26] Onicescu O. (1966). Énergie informationnelle. C. R. Acad. Sci. Ser. A.

[27] Beckner W. (1975). Inequalities in Fourier analysis on *R^n^*. Proc. Natl. Acad. Sci. USA.

[28] Rajagopal A.K. (1995). The Sobolev inequality and the Tsallis entropic uncertainty relation. Phys. Lett. A.

[29] Białynicki-Birula I. (2006). Formulation of the uncertainty relations in terms of the Rényi entropies. Phys. Rev. A.

[30] Zozor S., Vignat C. (2007). On classes of non-Gaussian asymptotic minimizers in entropic uncertainty principles. Phys. A.

[31] Olendski O. (2019). Rényi and Tsallis entropies: three analytic examples. Eur. J. Phys..

[32] Jizba P., Arimitsu T. (2004). The world according to Rényi: thermodynamics of multifractal systems. Ann. Phys. (N.Y.).

[33] Jizba P., Dunningham J.A., Joo J. (2015). Role of information theoretic uncertainty relations in quantum theory. Ann. Phys. (N.Y.).

[34] Tsallis C. (2009). Introduction to Nonextensive Statistical Mechanics.

[35] Tsallis C. (2011). The nonadditive entropy *S_q_* and its applications in physics and elsewhere: some remarks. Entropy.

[36] Geilikman M.B., Golubeva T.V., Pisarenko V.F. (1990). Multifractal patterns of seismicity. Earth Planet. Sci. Lett..

[37] Carranza M.L., Acosta A., Ricotta C. (2007). Analyzing landscape diversity in time: the use of Rényi’s generalized entropy function. Ecol. Indic..

[38] Rocchini D., Delucchi L., Bacaro G., Cavallini P., Feilhauer H., Foody G.M., He K.S., Nagendra H., Porta C., Ricotta C. (2013). Calculating landscape diversity with information-theory based indices: A GRASS GIS solution. Ecol. Inf..

[39] Drius M., Malavasi M., Acosta A.T.R., Ricotta C., Carranza M.L. (2013). Boundary-based analysis for the assessment of coastal dune landscape integrity over time. Appl. Geogr..

[40] Rosso O.A., Martin M.T., Figliola A., Keller K., Plastino A. (2006). EEG analysis using wavelet-based information tools. J. Neurosci. Meth..

[41] Tozzi A., Peters J.F., Çankaya M.N. (2018). The informational entropy endowed in cortical oscillations. Cogn. Neurodyn..

[42] Costa M., Goldberger A.L., Peng C.-K. (2005). Multiscale entropy analysis of biological signals. Phys. Rev. E.

[43] Aptekarev A.I., Dehesa J.S., Sánchez-Moreno P., Tulyakov D.N. (2012). Rényi entropy of the infinite well potential in momentum space and Dirichlet-like trigonometric functionals. J. Math. Chem..

[44] Toranzo I.V., Dehesa J.S. (2016). Rényi, Shannon and Tsallis entropies of Rydberg hydrogenic systems. Europhys. Lett..

[45] Dehesa J.S., Toranzo I.V., Puertas-Centeno D. (2016). Entropic measures of Rydberg-like harmonic states. Int. J. Quantum Chem..

[46] Aptekarev A.I., Tulyakov D.N., Toranzo I.V., Dehesa J.S. (2016). Rényi entropies of the highly-excited states of multidimensional harmonic oscillators by use of strong Laguerre asymptotics. Eur. Phys. J. B.

[47] Nasser I., Zeama M., Abdel-Hady A. (2017). The Rényi entropy, a comparative study for He-like atoms using the exponential-cosine screened Coulomb potential. Results Phys..

[48] Mukherjee N., Roy A.K. (2018). Information-entropic measures in free and confined hydrogen atom. Int. J. Quantum Chem..

[49] Mukherjee N., Roy A.K. (2018). Information-entropic measures in confined isotropic harmonic oscillator. Adv. Theory Simul..

[50] Ou J.-H., Ho Y.K. (2019). Benchmark calculations of Rényi, Tsallis entropies, and Onicescu information energy for ground state helium using correlated Hylleraas wave functions. Int. J. Quantum Chem..

[51] Zeama M., Nasser I. (2019). Tsallis entropy calculation for non-Coulombic helium. Physica A.

[52] Ou J.-H., Ho Y.K. (2019). Shannon, Rényi, Tsallis entropies and Onicescu information energy for low-lying singly excited states of helium. Atoms.

[53] Klebanov I.R., Pufu S.S., Sachdev S., Safdi B.R. (2012). Rényi entropies for free field theories. J. High Energy Phys..

[54] Chen B., Zhang J. (2013). On short interval expansion of Rényi entropy. J. High Energy Phys..

[55] Dong X. (2016). The gravity dual of Rényi entropy. Nature Commun..

[56] Islam R., Ma R., Preiss P.M., Tai M.E., Lukin A., Rispoli M., Greiner M. (2015). Measuring entanglement entropy in a quantum many-body system. Nature.

[57] Kaufman A.M., Tai M.E., Lukin A., Rispoli M., Schittko R., Preiss P.M., Greiner M. (2016). Quantum thermalization through entanglement in an isolated many-body system. Science.

[58] Brydges T., Elben A., Jurcevic P., Vermersch B., Maier C., Lanyon B.P., Zoller P., Blatt R., Roos C.F. (2019). Probing Rényi entanglement entropy via randomized measurements. Science.

[59] Prudnikov A.P., Brychkov Y.A., Marichev O.I. (1992). Integrals and Series.

[60] Fikhtengol’ts G.M. (1965). The Fundamentals of Mathematical Analysis.

[61] Wehner S., Winter A. (2010). Entropic uncertainty relations—A survey. New J. Phys..

[62] Coles P.J., Berta M., Tomamichel M., Wehner S. (2017). Entropic uncertainty relations and their applications. Rev. Mod. Phys..

[63] De Palma G., Trevisan D., Giovannetti V., Ambrosio L. (2018). Gaussian optimizers for entropic inequalities in quantum information. J. Math. Phys..

[64] Aharonov Y., Bohm D. (1961). Further considerations on electromagnetic potentials in the quantum theory. Phys. Rev..

[65] Merzbacher E. (1962). Single valuedness of wave fucntions. Am. J. Phys..

[66] Feĭnberg E.L. (1962). Ob “osoboi” roli elektromagnitnych potencialov v kvantovoi mechanike. Usp. Fiz. Nauk.

[67] Peshkin M. (1981). Aharonov-Bohm effect in bound states: theoretical and experimental status. Phys. Rev. A.

[68] Tinoco I., Woody R.W. (1964). Optical rotation of oriented helices. IV. A free electron on a helix. J. Chem. Phys..

[69] Kibis O.V., Malevannyy S.V., Huggett L., Parfitt D.G.W., Portnoi M.E. (2005). Superlattice properties of helical nanostructures in a transverse electric field. Electromagnetics.

[70] Vorobyova J.S., Vorob’ev A.B., Prinz V.Y., Toropov A.I., Maude D.K. (2015). Magnetotransport in two- dimensional electron gas in helical nanomembranes. Nano Lett..

[71] Downing C.A., Robinson M.G., Portnoi M.E. (2016). Nanohelices as superlattices: Bloch oscillations and electric dipole transitions. Phys. Rev. B.

[72] Antolín J., López-Rosa S., Angulo J.C. (2009). Renyi complexities and information planes: Atomic structure in conjugated spaces. Chem. Phys. Lett..

[73] Nath D., Chosh P. (2019). A generalized statistical complexity based on Rényi entropy of a noncommutative anisotropic oscillator in a homogeneous magnetic field. Int. J. Mod. Phys. A.

[74] Coles P.J., Katariya V., Lloyd S., Marvian I., Wilde M.M. (2019). Entropic energy-time uncertainty relation. Phys. Rev. Lett..

[75] Rastegin A.E. (2019). On entropic uncertainty relations for measurements of energy and its “complement”. Ann. Phys..

